# Supplementary material for: The Associations of Meteorological and Environmental Factors with Memory Function of the Older Age in Urban Areas
Source: Int J Environ Res Public Health. 2022 Apr 30;19(9):5484. doi: 10.3390/ijerph19095484 (PMC9105547; doi:10.3390/ijerph19095484)
Supplement: Supplementary file 1 [file ijerph-19-05484-s001.zip › ijerph-1649124-supplementary.pdf]

Supplementary materials

As shown in Figure S1 and Table S1, we checked the statistical assumptions, the results showed that the samples in this study conformed to the normal distribution, and there was not strong multicollinearity among those variables.

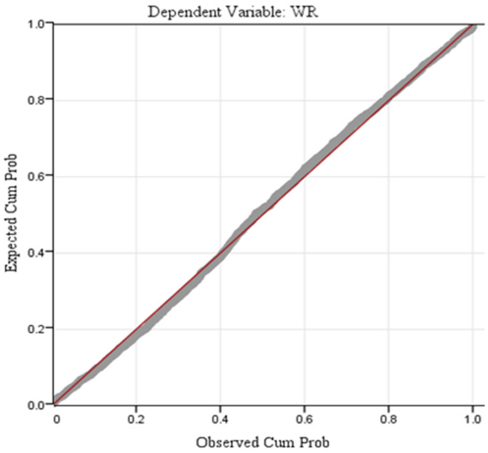

Figure S1. Normal P-P plot of regression standardized residual.

Table S1. Analysis of multicollinearity by stepwise regression.

Variable	Collinearity Statistics		
	Beta	Tolerance	VIF
Age	−0.108	0.995	1.005
Gender	0.298	0.995	1.005
Chronic disease	−0.007	0.993	1.007
Temperature	0.020	0.818	1.222
Humidity	−0.048	1.000	1.000
Greening	−0.029	0.911	1.098
